# Size-exclusion chromatography can identify faster-associating protein complexes and evaluate design strategies

**DOI:** 10.1186/1756-0500-2-135

**Published:** 2009-07-15

**Authors:** Chad L Mayer, W Kalani Snyder, Monika A Swietlicka, Andrew D VanSchoiack, Chad R Austin, Benjamin J McFarland

**Affiliations:** 1 Department of Chemistry and Biochemistry, Seattle Pacific University, 3307 Third Avenue West, Seattle, WA 98119-1997, USA; 2 Current address : Seattle Biomedical Research Institute, Seattle, WA 98109, USA; 3 Current address : Dept. of Microbiology, University of Colorado – Denver, Aurora, CO 80045, USA

## Abstract

**Background:**

We previously developed a set of rationally designed mutant MICA protein ligands for the NKG2D immunoreceptor in which MICA was mutated at residues that do not contact NKG2D. Some of these MICA mutants, predicted by RosettaDesign to be destabilized, bound NKG2D with affinities enhanced by more than an order of magnitude when evaluated by surface plasmon resonance (SPR).

**Findings:**

Small-zone size-exclusion chromatography (SEC) detected persistent high-affinity MICA mutant-NKG2D complexes in solution as early-eluting peaks. The SEC binding assay used standard protein purification instrumentation to evaluate complex stability, qualitatively paralleled the SPR results, and successfully discriminated among complexes that differed only in on-rates. We used the SEC binding assay, along with SPR, to assess the results of a follow-up design strategy targeting the non-interfacial redesigned region. Both SEC and SPR agreed that these mutations did not enhance affinity as much as previous mutants. When the SEC binding assay was run in 1 M urea, only the highest affinity complex was detected.

**Conclusion:**

This SEC binding assay provides a correlation with SPR results for protein complex affinities, detecting changes in complex on-rates, and tunable to lower sensitivity with 1 M urea. The SEC binding assay is complementary to other protein design evaluation methods, can be adapted to the undergraduate research laboratory, and may provide additional structural information about changes in hydrodynamic radii from elution times. Our assay allowed us to conclude that further alteration of MICA at non-contacting residues is unlikely to further enhance NKG2D affinity.

## Background

Several protein design algorithms have been produced over the past decade for rationally altering and optimizing the cores of proteins, protein-ligand interfaces, and protein-protein interfaces for structural and therapeutic application [1], including RosettaDesign [2]. Protein design schemes require a step for screening a set of candidate proteins. Size-exclusion chromatography (SEC) is an option for design evaluation if a protein's size is changed significantly or a larger, high-affinity protein-protein complex is formed. For protein-protein complexes, the SEC column can be saturated with protein for a large-zone assay [3], or a small plug of protein can be injected onto a column for a small-zone assay [4,5]. Small-zone techniques have been used for the MICA-NKG2D protein-protein interaction we investigate in this paper using small (analytical) amounts of protein and detecting the presence or absence of persistent complexes [6], as a purification step for MICA-NKG2D complex crystallization [7], and for diverse ends with other proteins [8-10].

SEC has the technical advantages of preparative scale, speed and cost-effectiveness relative to binding techniques such as surface plasmon resonance (SPR). In addition, persistence of a complex through an SEC column implies that the proteins physically adhered or equilibrated quickly enough to effectively adhere on a scale of minutes to hours, even as unbound molecules were partitioned away by the action of the column. This physical evidence that the protein-protein complex has been maintained over a long time shows that the complex has a significant "residence time," a concept which has proved useful for small-molecule drugs [11] and may be useful for finding or characterizing potent designed-protein drugs or receptor-ligand complexes [12].

**Figure 1:**
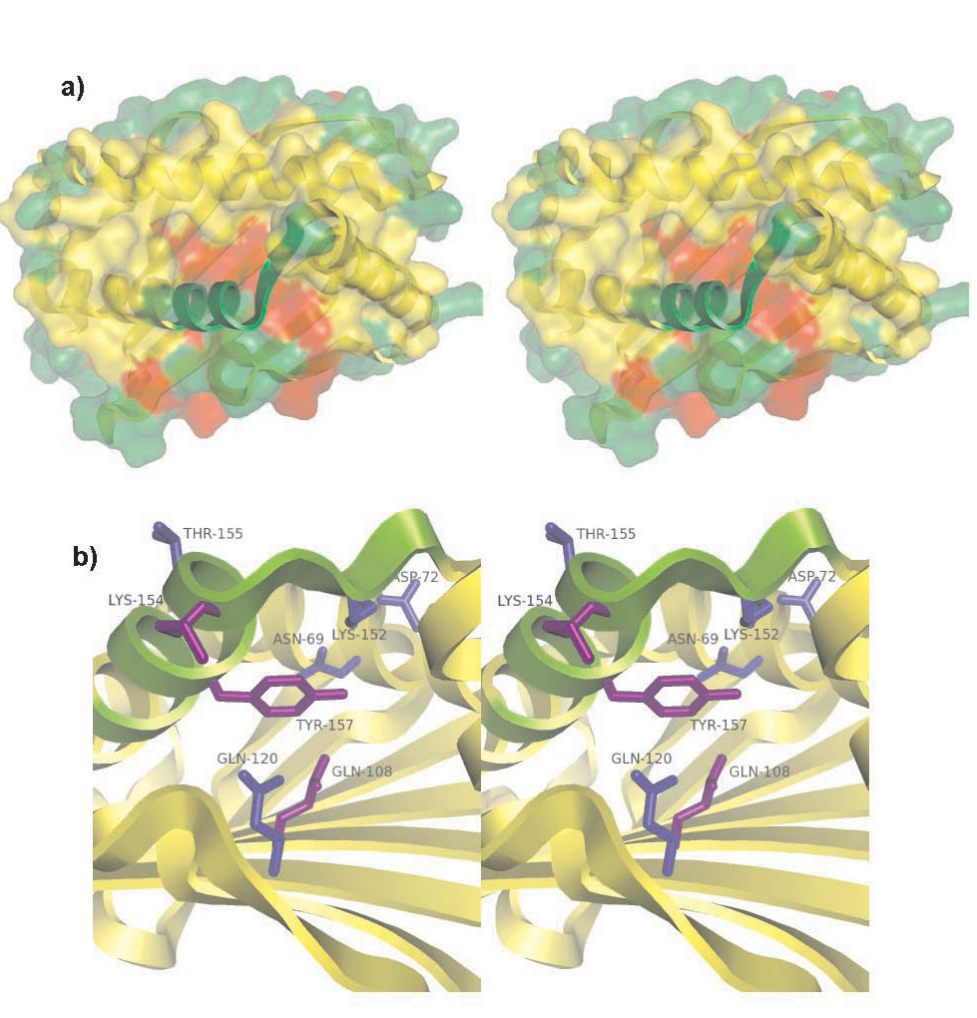
**Location of disordered region and design mutations in the MICA structure**. (a) The receptor-bound structure of MICA (Protein Data Bank ID 1HYR chain C) in stereo, shown as ribbons and colored by PDB-assigned secondary structure of unbound MICA (PDB ID 1B3J), with beta sheets red, helices yellow and loops green. (Residues in the α2 helix of receptor-bound MICA that are absent from the crystal structure of unbound MICA are colored green.) The solvent-accessible surface of unbound MICA is overlaid on the receptor-bound structure with the same coloring, showing how the disordered region exposes the underlying beta-sheet. (b) Side view in stereo of the receptor-bound wild-type MICA structure used by RosettaDesign, with the helix backbone that is not observed in the unbound structure colored green. The eight redesigned residues are shown as sticks and labeled. The residues mutated in only the first design strategy, summarized in Table 1, are colored blue, and the residues that were also mutated in the second design strategy, summarized in Table 2, are colored purple. Figures made with PyMol .

Here we describe the full results a small-zone analytical SEC protein-protein binding assay developed to probe the design space for the MICA-NKG2D interaction, which was mentioned but not detailed in a previous publication [13]. MICA binds homodimeric NKG2D on the surface of certain immunocytes, triggering the death of stressed cells such as transformed cancerous cells [14]. We previously described how a set of 25 mutant MICA proteins was made in which eight MICA residues were varied [13]. These mutations were not located directly at the NKG2D-MICA binding interface, but were below it, located within and under the α2 helix (Figure 1). This atypical design strategy produced unexpected results: MICA proteins with lower design scores, predicting more stable MICA proteins, bound NKG2D with the same or weaker affinity, while some MICA proteins with higher design scores, predicting that the MICA proteins themselves would be less stable, bound NKG2D with greater affinity [13]. Here we report the complete results of our SEC binding assay and correlate them with the previously reported thermodynamic and kinetic results [13] obtained by surface plasmon resonance (SPR). We also extend the data set with follow-up MICA designs, bringing to conclusion our analysis of destabilizing design strategies.

## Results

### SEC binding assays can detect stabilized protein complexes in the micromolar-to-nanomolar range

**Figure 2:**
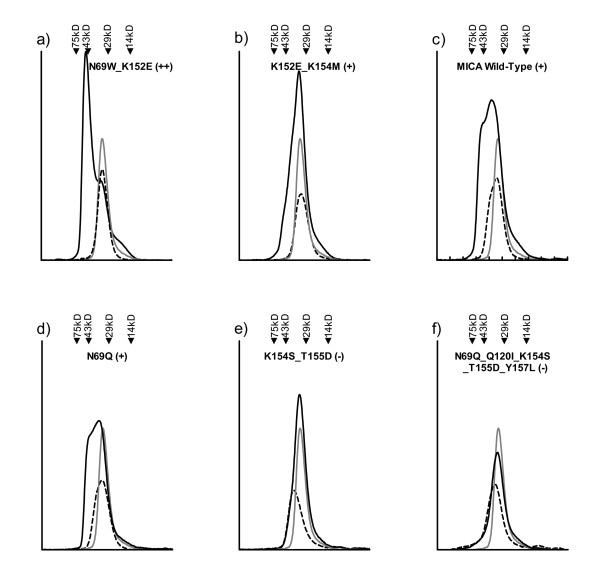
**Differences among NKG2D-mutant MICA complexes as observed in the SEC binding assay**. The elution profile of UV absorbance vs. time as constant flow resulting from an injection of NKG2D alone is shown as a gray line; MICA mutant or wild-type alone is shown as a dashed line; and NKG2D and MICA mutant or wild-type mixed in 2:1 molar ratios is shown as a solid line. The elution times of size standards under identical conditions are shown above each graph. Graphs are labelled with the specific representative mutant. (a) Some mutants elute as a ~60-kDa peak (++). (b-d) Some MICA mutants and wild-type elute with an early shoulder undifferentiated from the ~30-kDa unbound peak (+). (e-f) Some MICA mutants with stabilizing mutations elute only as a ~30-kDa peak aligned with the elution time of both proteins alone (-).

Steinle *et al*. observed that recombinantly produced and refolded NKG2D, mixed with wild-type MICA and injected onto an SEC column, eluted as an early shoulder to the unbound protein peak, at a molecular weight corresponding to a ~60 kDa complex rather than its individual ~30 kDa components. [6] We observed the same results with our preparations of wild-type MICA mixed with homodimeric NKG2D (Figure 2c), and repeated these conditions for our set of redesigned MICA mutants (Figure 2). Some MICA mutants with high design scores (predicting MICA destabilization) mixed in a 1:2 molar ratio with NKG2D eluted as a separate, early ~60 kDa peak (Figure 2a). When this early peak was collected and analyzed by reducing SDS-PAGE, protein bands were observed at the molecular weights corresponding to both MICA (30 kDa) and NKG2D (15 kDa). Other MICA mutants mixed with NKG2D eluted as early shoulders to the 30-kDa peak (Figure 2b and 2d), or as symmetrical ~30-kDa peaks (Figure 2e and 2f). A qualitative correlation can be observed between SPR equilibrium ΔG of binding and SEC bind assay results: Observation of an early shoulder corresponds to a low-micromolar range of affinity like wild-type MICA binding NKG2D. A differentiated early peak corresponds to high-nanomolar binding, as with MIC_N69W_K152E, and the absence of an early peak corresponds to low-to-mid-micromolar binding, as with MIC_N69Q_Q120I_K154S_T155D_Y157L.

### SEC binding assays can detect differences in protein-protein on-rates

**Figure 3:**
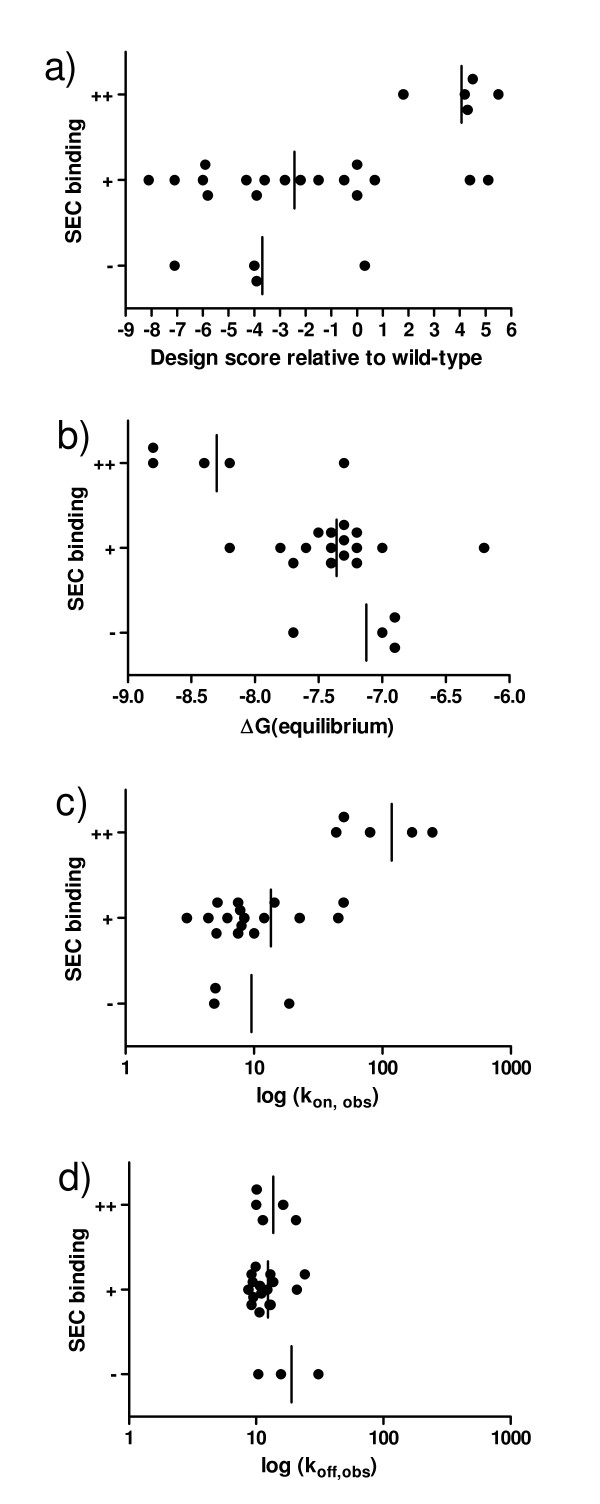
**Comparison of SPR and SEC binding assays for evaluation of redesigned MICA proteins**. Each MICA mutant from Table 1 is organized according to the results of the SEC binding assay and graphed against a different parameter. The average of each category is shown as a vertical line. (a) SEC results compared to RosettaDesign score [13] shows that negative RosettaDesign scores (predicted stabilized) only have binding results similar to or worse than wild-type, while positive RosettaDesign scores (predicted destabilized) include all 5 MICA mutants found to bind more tightly than wild-type. SEC results compared to SPR results [13] from (b) equilibrium and (c-d) kinetic analysis show similar correlations between equilibrium affinity and on-rate with the highest-affinity mutants by SEC, while no correlation with off-rate is seen because the set of mutants does not vary significantly in this parameter. Observed k_on _and k_off _rates from single-step kinetic fits for mutants predicted to be stabilized, and from two-step kinetic fits for mutants predicted to be destabilized (k_+1 _and k_-2_, respectively). (P values for the difference between ++ and + complexes: t-test P = 0.0013 for Figure 3a; 0.0006 for Figure 3b; 0.0001 for Figure 3c; and 0.6 for Figure 3d.)

The SPR study showed that some high design scores are associated with high affinity, and the SEC binding assay leads to the same conclusion (Figure 3). Equilibrium affinity agrees with the sets of protein binding strengths described by the SEC binding assay. Our design strategy altered non-contacting residues that did not change the off-rates, but did cause a large difference in on-rates. The SEC binding assay detected this, discriminating among complexes that had significantly varying on-rates, and the off-rates for this set of proteins were so similar that they were not a significant factor.

### Rationally designed MICA destabilization at single non-contacting residues does not enhance NKG2D binding

**Figure 4:**
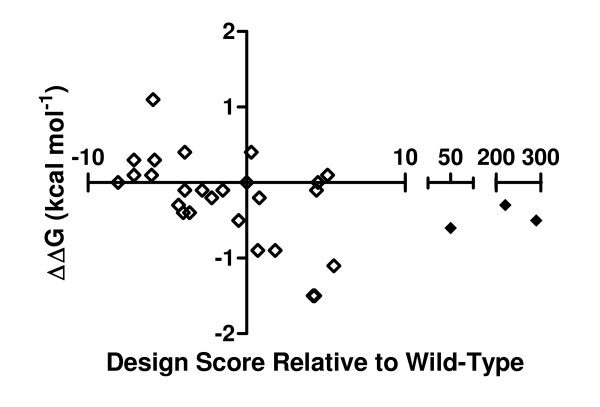
**Comparison of the initial and follow-up design strategies**. The difference in free energy of NKG2D binding relative to wild-type MICA is shown for each MICA mutant tested by SPR, from affinities determined at equilibrium. The RosettaDesign score relative to wild-type is plotted on the x-axis. MICA mutants in the initial design set (internal stabilization or destabilization; Table 1) are shown as open diamonds. MICA mutants from the follow-up design set (significant internal destabilization; Table 2) are shown as filled diamonds.

A plot of NKG2D affinity vs. design score for the initial set of 25 MICA mutants produced previously shows that an area of design space was unfilled because the initial design strategy was biased toward stabilizing mutations and no MICA mutant was destabilized by more than 6 units. (Figure 4) After we found that mild MICA destabilization increased NKG2D affinity by more than an order of magnitude (Table 1), we then designed three more destabilizing mutants at the same locations to confirm if we were past the point of "diminishing returns," at which increased MICA destabilization would destabilize the complex with NKG2D. Using RosettaDesign, non-cysteine residues were modeled at residues 108, 154, and 157. Point mutations predicted to provide a range of large disruptions to the wild-type structure were selected from the candidate designs produced by Rosetta, and prepared as previously reported [13]. In the SEC binding assay, none bound NKG2D better than wild-type MICA (Table 2). SPR equilibrium analysis showed their affinities were not significantly enhanced (Figure 4).

**Table 1:** SEC evaluation of predicted stabilized or mildly destabilized MICA mutants

MIC Mutant	Design Score	SEC Binding
N69Q_Q108L_Q120I_K154S_T155D	-8.1	+
N69Q_Q120I_K154S_T155D_Y157L	-7.1	-
N69Q_D72F_K154S_T155D	-7.1	+
N69Q_Q120I_K154S_T155D	-6	+
N69Q_D72F_Q108L_K152V_Y157L	-5.9	+
K152V_K154S_T155D_Y157L	-5.8	+
N69Q_K154D	-4.3	+
K154S_T155D	-4	-
K152V_K154S_T155D	-3.9	-
N69Q_D72F_Q108L	-3.9	+
N69Q_D72F	-3.6	+
N69Q	-2.8	+
K152V_Y157L	-2.2	+
K154D	-1.5	+
Q108L	-0.5	+
Wild-type	0	+
D72W	0.3	-
Q120I	0.7	+
Q120I_K154M	0.8	n/a
N69W	1.8	++
N69W_K152E_K154S	4.2	++
N69W_K152E_K154D	4.3	++
K152E_K154M	4.4	+
N69W_D72F_K152E	4.5	++
N69W_D72W_K152E	5.1	+
N69W_K152E	5.5	++

Design scores are listed relative to wild-type. Results of SEC binding assay follow the examples in Figure 2; n/a, not applicable because unbound mutant did not elute from column due to non-specific binding.

**Table 2:** SEC and SPR evaluation of predicted significantly destabilized MICA point mutants

				Buffer + 0 M Urea		
						
				Elution	Apparent	Apparent
MIC Mutant	Design Score	ΔG_eq_ (kcal mol^-1^)	SEC Binding	Volume (mL)	MW (kDa)	r (Å)
WT	0	-7.3 ± 0.1	+	3.5 ± 0.1	30 ± 2	23 ± 2
N69W_K152E_K154D	4	-8.8 ± 0.1	++	3.4 ± 0.1	30 ± 2	23 ± 2
Y157W	50	-7.9 ± 0.1	-	3.6 ± 0.1	30 ± 2	23 ± 2
K154P	220	-7.6 ± 0.1	+	3.1 ± 0.1	34 ± 2	25 ± 2
Q108W	290	-7.8 ± 0.2	+	3.1 ± 0.1	34 ± 2	25 ± 2

				Buffer + 1 M Urea		
				Elution	Apparent	Apparent
MIC Mutant			SEC Binding	Volume (mL)	MW (kDa)	r (Å)

WT			-	2.8 ± 0.1	38 ± 2	27 ± 3
N69W_K152E_K154D			++	2.9 ± 0.1	37 ± 2	26 ± 3
Y157W			-	2.8 ± 0.1	39 ± 2	27 ± 3
K154P			-	2.6 ± 0.1	41 ± 3	28 ± 3
Q108W			-	2.7 ± 0.1	40 ± 3	28 ± 3

Design scores are listed relative to wild-type. ΔG values determined at equilibrium by SPR. Wild-type and high-affinity MIC N69W_K152E_K154D SPR values as reported previously in [13]. Upper table shows SEC results with no added urea, and lower table shows SEC results in the presence of 1 M additional urea. Results of SEC binding assay follow the examples in Figure 2. Elution volume measured relative to the elution time of blue dextran. Apparent molecular weight (MW) and radii (r) determined by line fit of elution time to Low Molecular Weight Standards (GE Healthcare). Standard errors are from triplicate experiments as described in Methods.

### Destabilized mutants elute early in analytical SEC

**Figure 5:**
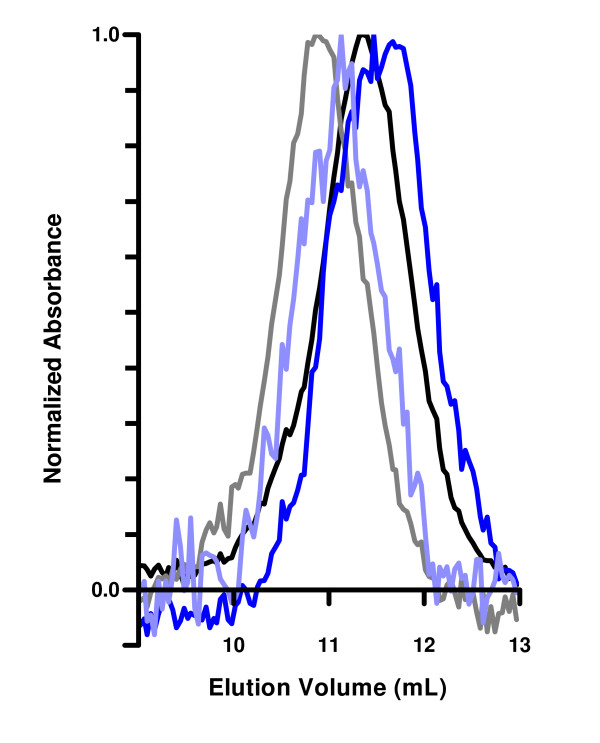
**Effect of 1 M urea on the elution profiles of MICA wild-type and MIC_Q108W**. Elution profiles of normalized UV absorbance vs. elution volume for injections of MICA wild-type (black) and MIC_Q108W (blue) in HBS-EA; and for MICA wild-type (gray) and MIC_Q108W (light blue) in HBS-EA with 1 M urea added.

In the SEC binding assay, the peak maximum of the unbound MICA mutant would decrease, moving from 3.5 mL for mutants with design scores of 50 or below to 3.1 mL for higher design scores (Figure 5), increasing apparent hydrodynamic radius from 23 Å to 25 Å (Table 2). When the column was saturated with buffer containing 1 M urea, the two mutants with the largest predicted destabilizations continued to elute early (Figure 5). The early-eluting shoulders for wild-type and the point-destabilized mutants were no longer observed. High-affinity MICN69W_K152E_K154D when mixed with NKG2D still eluted in 1 M urea as an early peak (Table 2).

## Discussion

In this study SEC formed the basis of a simple binding assay to assess protein-protein interaction strength, complementary to SPR binding assays. SEC results alone could evaluate the qualitative success or failure of exploring these regions of design space, although without quantitative kinetic or thermodynamic detail. The correlation of SEC and SPR is similar to the correlation between SEC and analytical ultracentrifugation (AUC) in that results from the two techniques generally agree, with SEC using common equipment, but AUC considered the "gold standard" [10,15-18]. AUC requires dedicated instrumentation, while SEC uses protein purification equipment and is faster [9]. SEC may be used to bring protein design to undergraduate research programs with limited equipment budgets, where the same pump used for protein chromatography during purification can be used for design evaluation through an SEC binding assay. The range of affinities that could be discriminated by the SEC binding assay was appropriate to design of the low-micromolar MICA-NKG2D interaction. Addition of 1 M urea to the assay tuned it to be less sensitive to lower-affinity binding, while still discriminating between our most successful design and other candidates.

The relationship between elution time and protein-protein affinity has been modelled for small-zone SEC emphasizing the role of the off-rate in determining complex persistence. [4,5] Our data show that SEC can discriminate among complexes of different affinities that primarily differ in on-rate. We hypothesize that a fast on-rate can allow a complex to re-attach before substantial separation, so that the complex remains in a small zone, even if the off-rate is fast.

Unbound mutant proteins with point mutations that are predicted to cause large destabilizations appear slightly larger than wild-type MICA by SEC. The average hydrodynamic radius of these proteins appears increased by destabilization; a similar observation using SEC-light scattering led to the conclusion that the protein in question was partially denatured [16]. For partially disordered proteins, the interplay between disorder and affinity is incompletely understood. Research into the relationship between dynamics and affinity may delineate which types of disorder promote binding, such as fly-casting [19,20] or ground-state destabilization [21], and which types of disorder inhibit binding.

## Conclusion

While the specific dynamic and structural impacts of the destabilizing mutations are only hinted at by the increased elution times of the SEC binding assay, the impact of these mutations on binding affinity is clear, in that none are stabilized in NKG2D affinity relative to wild-type MICA by more than 0.5 kcal/mol, and none persist through the column as a bound complex. We decided from these results to target other regions for future design. The persistence of a high-affinity complex through the column even in the presence of 1 M urea could imply significantly increased persistence time in the biological environment as well.

## Methods

### Protein design and production

The set of mutants in Table 1 was designed and produced as described previously [13]. Because the initial design strategy focused on increasing the stability of MICA in the region of the disordered loop, only 603 of 4608 designs were predicted to destabilize the receptor-bound conformation relative to wild-type. The set of mutants in Table 2 was designed to produce more destabilizing mutants. RosettaDesign [2] v2.0 was used with the coordinates of receptor-bound MICA from the NKG2D-MICA crystal structure (PDB ID 1HYR) [7]. Three of the eight previously altered locations at the center of the disordered region located on different structural elements were chosen (Figure 1b). RosettaDesign was used to model the results of mutating of each of these three residues to the 19 non-cysteine amino acids with the backbone fixed to the receptor-bound coordinates. Mutations with large positive scores relative to wild-type were chosen for analysis.

Proteins were produced as described previously [13]. Before use in binding assays the proteins were dialyzed into HBS-EA buffer (10 mM HEPES [pH 7.4], 150 mM NaCl, 3 mM EDTA, and 0.02% sodium azide). Protein concentrations were determined by Nanodrop ND-1000 (Thermo Scientific) absorbance at 280 nm and by bicinchoninic (BCA) assay (Thermo Scientific).

### SEC binding assays

20 μmol each of homodimeric NKG2D and the MIC-A mutant were combined with HBS-EA buffer to a volume of 300 μL, mixed and immediately injected onto a Superdex 75 10/300 gel-filtration column (GE Healthcare) with a 500-μL loop at 0.5 mL/min using an AKTA PrimePlus FPLC system (GE Healthcare). The eluting protein was detected by UV absorbance at 280 nm. These were compared to injections of 20 μmol of homodimeric NKG2D or MICA alone. Columns were calibrated with Gel Filtration Calibration LMW standards (GE Healthcare: conalbumin, ovalbumin, carbonic anhydrase, and ribonuclease A, and blue dextran for void volume determination; also Sigma: cytochrome c). Several individual proteins and mixed receptor-ligand complexes were injected three or more times onto the same column, resulting in variations in elution volume of no more than 0.1 mL. Fractions were collected using the AKTA Prime fraction collector for analysis by reducing analytical SDS-PAGE using Coomassie Blue staining. Observed variations in elution volume for triplicate experiments with urea in the buffer were as listed in Table 2.

### SPR binding assays

For the first set of mutants (Table 1), determination of NKG2D-mutant MICA kinetics and thermodynamics by SPR was previously described [13]. NKG2D affinities for the second set of MICA mutants (Table 2) were determined by equilibrium binding analysis as used in the previous study. (Fast kinetics precluded the use of kinetic fits.) Standard errors reported in the data tables result from triplicate (or more) experiments.

## Competing interests

The authors declare that they have no competing interests.

## Authors' contributions

CLM and WKS carried out SEC binding assays. WKS and BJM carried out SPR binding assays. CLM, WKS, MAS, ADV, and CRA designed, made and purified mutant proteins. All authors participated in interpretation of the data. CLM, WKS, CRA and BJM drafted the manuscript. All authors have read and approved the final manuscript.

## References

[B1] Kortemme T, Baker D (2004). Computational design of protein-protein interactions. Curr Opin Chem Biol.

[B2] Kortemme T, Baker D (2002). A simple physical model for binding energy hot spots in protein-protein complexes. Proceedings of the National Academy of Sciences of the United States of America.

[B3] Winzor DJ, Harding SE, Chowdhry BZ (2001). Quantitative characterization of ligand binding by chromatography. Protein-Ligand Interactions: Hydrodynamics and Calorimetry: A Practical Approach.

[B4] Stevens FJ (1989). Analysis of protein-protein interaction by simulation of small-zone size exclusion chromatography. Stochastic formulation of kinetic rate contributions to observed high-performance liquid chromatography elution characteristics. Biophys J.

[B5] Wilton R, Myatt EA, Stevens FJ, Fu H (2004). Analysis of protein-protein interactions by simulation of small-zone gel filtration chromatography. Protein-Protein Interactions: Methods and Protocols.

[B6] Steinle A, Li P, Morris DL, Groh V, Lanier LL, Strong RK, Spies T (2001). Interactions of human NKG2D with its ligands MICA, MICB, and homologs of the mouse RAE-1 protein family. Immunogenetics.

[B7] Li P, Morris DL, Willcox BE, Steinle A, Spies T, Strong RK (2001). Complex structure of the activating immunoreceptor NKG2D and its MHC class I-like ligand MICA. Nat Immunol.

[B8] Franzini M, Bramanti E, Ottaviano V, Ghiri E, Scatena F, Barsacchi R, Pompella A, Donato L, Emdin M, Paolicchi A (2008). A high performance gel filtration chromatography method for gamma-glutamyltransferase fraction analysis. Anal Biochem.

[B9] le Maire M, Arnou B, Olesen C, Georgin D, Ebel C, Moller JV (2008). Gel chromatography and analytical ultracentrifugation to determine the extent of detergent binding and aggregation, and Stokes radius of membrane proteins using sarcoplasmic reticulum Ca2+-ATPase as an example. Nat Protoc.

[B10] Gralle M, Oliveira CL, Guerreiro LH, McKinstry WJ, Galatis D, Masters CL, Cappai R, Parker MW, Ramos CH, Torriani I (2006). Solution conformation and heparin-induced dimerization of the full-length extracellular domain of the human amyloid precursor protein. J Mol Biol.

[B11] Copeland RA, Pompliano DL, Meek TD (2006). Drug-target residence time and its implications for lead optimization. Nat Rev Drug Discov.

[B12] Tummino PJ, Copeland RA (2008). Residence time of receptor-ligand complexes and its effect on biological function. Biochemistry.

[B13] Lengyel CS, Willis LJ, Mann P, Baker D, Kortemme T, Strong RK, McFarland BJ (2007). Mutations designed to destabilize the receptor-bound conformation increase MICA-NKG2D association rate and affinity. J Biol Chem.

[B14] Strong RK, McFarland BJ (2004). NKG2D and Related Immunoreceptors. Adv Protein Chem.

[B15] Berkowitz SA (2006). Role of analytical ultracentrifugation in assessing the aggregation of protein biopharmaceuticals. AAPS J.

[B16] Philo JS (2006). Is any measurement method optimal for all aggregate sizes and types?. AAPS J.

[B17] Gualfetti PJ, Iwakura M, Lee JC, Kihara H, Bilsel O, Zitzewitz JA, Matthews CR (1999). Apparent radii of the native, stable intermediates and unfolded conformers of the alpha-subunit of tryptophan synthase from E. coli, a TIM barrel protein. Biochemistry.

[B18] Gabrielson JP, Brader ML, Pekar AH, Mathis KB, Winter G, Carpenter JF, Randolph TW (2007). Quantitation of aggregate levels in a recombinant humanized monoclonal antibody formulation by size-exclusion chromatography, asymmetrical flow field flow fractionation, and sedimentation velocity. J Pharm Sci.

[B19] Hoffman RM, Blumenschein TM, Sykes BD (2006). An interplay between protein disorder and structure confers the Ca2+ regulation of striated muscle. J Mol Biol.

[B20] Shoemaker BA, Portman JJ, Wolynes PG (2000). Speeding molecular recognition by using the folding funnel: the fly-casting mechanism. Proceedings of the National Academy of Sciences of the United States of America.

[B21] Horn JR, Kraybill B, Petro EJ, Coales SJ, Morrow JA, Hamuro Y, Kossiakoff AA (2006). The role of protein dynamics in increasing binding affinity for an engineered protein-protein interaction established by H/D exchange mass spectrometry. Biochemistry.

